# Protocol for a minigene splice assay using the pET01 vector

**DOI:** 10.1016/j.xpro.2025.103908

**Published:** 2025-06-18

**Authors:** Hannah Andreae, Marialessandra Curcio, Daniel Owrang, Sahar Esmaeelpour, Friederike Jahnke, Fritz Benseler, Nils Brose, Barbara Vona

**Affiliations:** 1 Institute of Human Genetics, University Medical Center Göttingen, 37073 Göttingen, Germany; 2 Institute for Auditory Neuroscience and InnerEarLab, University Medical Center Göttingen, 37073 Göttingen, Germany; 3 Auditory Neuroscience and Optogenetics Laboratory, German Primate Center, 37077 Göttingen, Germany; 4 Department of Molecular Neurobiology, Max Planck Institute for Multidisciplinary Sciences, 37075 Göttingen, Germany; 5 Department of Molecular Medicine and Medical Biotechnology, University of Naples Federico II, 80131 Naples, Italy

**Keywords:** Cell-based Assays, Genetics, Genomics, Sequencing, Molecular Biology

## Abstract

Aberrant splicing plays a major role in hereditary disorders, yet characterizing molecular effects of splice variants poses challenges. Here, we present a protocol for an *in vitro* minigene splice assay using the pET01 vector. We describe steps for assay design, minigene plasmid cloning, transfection, RNA isolation, and cDNA synthesis. We also detail procedures for quantitative capillary electrophoresis and optional subcloning. This protocol is useful when patient RNA is unavailable or target genes or isoforms are not expressed in accessible tissues.

## Highlights

• Protocol for a minigene splice assay using the pET01 vector
• Detailed steps on assay design, cloning, transfection, and RT-PCR
• Instructions on quantification using capillary electrophoresis

## Abstract (editor highlights)

Publisher’s note: Undertaking any experimental protocol requires adherence to local institutional guidelines for laboratory safety and ethics.

## Graphical abstract

**Figure:**
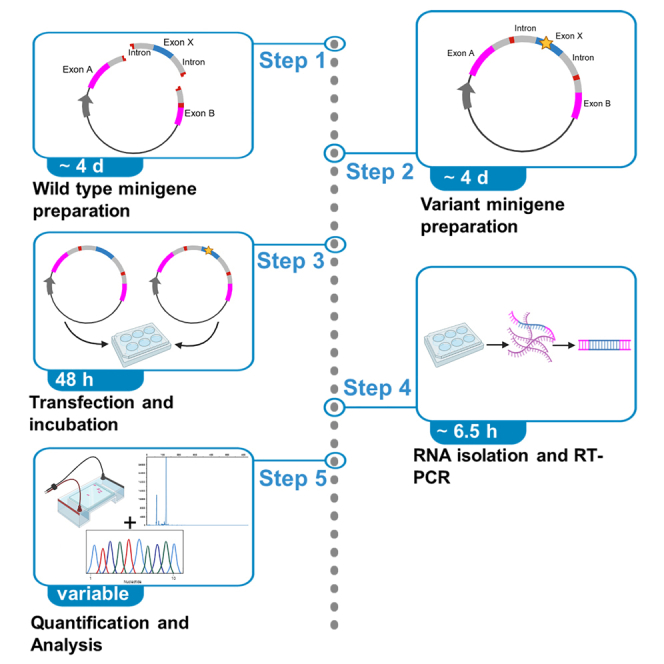

## Before you begin

An important effect of nucleotide sequence variations is their potential to significantly alter the splicing signal landscape utilized by the spliceosome. This is particularly relevant when analyses are solely conducted at the DNA level without incorporating RNA studies.^1^^,^^2^

In our investigation of genetic variants in otoferlin (*OTOF*), a protein expressed exclusively in the cochlea and brain,^3^ we found no practical methods to characterize splicing effects using commonly available patient tissues, such as blood or fibroblasts. We describe here an *in vitro* minigene splice assay that resolves this issue and provides an effective approach to elucidate spliceogenic effects in the absence of target tissues. Based on this method, we present data for four *OTOF* variants reported in patients, detailed in the expected results section and in supplementary material.

**Figure 1:**
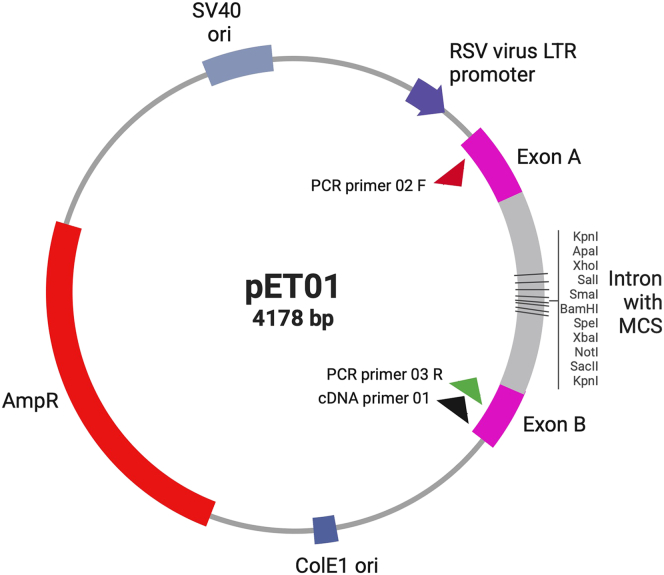
pET01 plasmid map pET01 plasmid map illustrating the multiple cloning site located within the intron, flanked by exons A and B, along with the primers used in this protocol aligned to the vector (adapted from datasheet “Exontrap” by MoBiTec GmbH^4^).

This protocol outlines the materials and methods required to functionally characterize genetic variants predicted to cause splicing aberrations using an *in vitro* (minigene) assay with the exon-trapping vector pET01 (MoBiTec GmbH, Göttingen, Germany). This vector contains an intrinsic splicing function, comprising a promoter followed by exon A, an intron with a multiple cloning site (into which the variant-containing region is cloned), exon B, and a sequence for the Poly-A tail (Figure 1). Upon transfection into eukaryotic cells, RNA expression and splicing of the mature RNA occurs (see datasheet “Exontrap” by MoBiTec GmbH^4^), allowing assessment of the variant impact on splicing that can be compared to the wild-type sequence.

In summary, we present an approach that provides a reproducible and controlled splicing environment for testing hypothetical or predicted splicing variants, which is particularly valuable when patient-derived RNA samples are unavailable or challenging to obtain.

### Institutional permissions (if applicable)

Before beginning this protocol, users must ensure that informed consent and ethical approval have been obtained for the use of patient-derived DNA. Institutional permission must be secured, if applicable. The example described here uses genomic DNA from a healthy adult volunteer, obtained with informed consent under protocols that did not require formal ethics committee approval. No identifiable information or clinical data were collected.

### Design assay-specific primers

**Timing:Variable**

Specific primers are necessary for amplifying the region containing the variant of interest, thereby creating the insert for pET01. In cases where patient DNA with the variant of interest is unavailable, primers for site-directed mutagenesis are required.

1. Design primers to amplify a large target region for cloning into pET01. The following steps exemplify a standard procedure for primer design.
  a. Select one or more exons, ensuring at least 60 base pairs (bp) of flanking introns.
    ***Note:*** We usually work with inserts ranging from 500−700 bp or more. The DNA sequence can be retrieved using Ensembl (www.ensembl.org/).
  b. The selected sequence is used as a source sequence in Primer3web (https://primer3.ut.ee).
    ***Note:*** Set the “Primer GC percentage” to a minimum of 47%, optimal at 50% and maximum of 53%. Select appropriate primers.
  c. Use SNPCheck (https://genetools.org/SNPCheck/snpcheck.htm) to detect single nucleotide polymorphisms (SNPs) ensuring optimal primer binding.
    **CRITICAL:** SNPs should be minimized or have a low minor allele frequency (e.g. <0.01).
  d. Perform multiple alignment queries against the human genome using UCSC Genome Browser Human BLAT Search tool (https://genome.ucsc.edu/cgi-bin/hgBlat).
  e. Identify two different restriction enzymes to use based on the multiple cloning sites (refer to the multiple cloning site of pET01 “Exontrap Handbook”^4^ or Figure 1) and incorporate the corresponding sites with three to four additional nucleotides at the 5′ end of the primers in the correct orientation.
  f. Confirm that no restriction sites are present within the insert sequence using the ApE (A plasmid Editor) application (https://jorgensen.biology.utah.edu/wayned/ape/).
    **CRITICAL:** Select two appropriate different restriction sites to be added to the forward and reverse primer respectively (e.g. ApaI, XhoI, SalI, SmaI, BamHI, SpeI, XbaI, NotI, SacII) to ensure correct orientation of the insert in the pET01 vector.
2. Design primers for site-directed mutagenesis with the NEBaseChanger online tool (https://nebasechanger.neb.com/).

***Note:*** All primers designed for the dataset provided with this work can be downloaded under Document S2.

### Other preparations

**Timing:24 h**

3. Seed the desired cell line into a 6-well plate at a density of 2 x 10^5^ cells/mL (approximately 400,000 cells per well) the day prior to transfection.

***Note:*** This protocol was optimized for HCT116 cells. For other cell lines, these conditions may vary.

4. Obtain the pET01 vector in advance.
  a. Rehydrate the vector according to the manufacturer’s instructions (5 μg lyophilized vector in 5 μl of dH_2_O).
  b. Transform 1 μl of the vector into *E. coli* according to standard lab protocols and plate the transformed bacteria on LB agar plates containing ampicillin to select for bacteria that received the plasmid.
    ***Note:*** A standard protocol is described in step 14.
  c. Pick a colony to inoculate a LB culture with ampicillin, incubate 16 h and isolate the plasmid.
    ***Note:*** A standard protocol is described in steps 28−32.
    **CRITICAL:** Make sure to store a back-up 16 h culture as a glycerol stock in −80°C.
    ***Note:*** For a comprehensive overview of pET01, we provide the full plasmid sequence with already marked primer and restriction sites. Download the document under Document S1.

## Key resources table

**Table:** 

REAGENT or RESOURCE	SOURCE	IDENTIFIER
**Bacterial and virus strains**		

NEB 5-alpha competent *E.coli* (high efficiency)	New England Biolabs	Cat# C2987H

**Biological samples**		

Healthy control genomic DNA sample	Healthy volunteer	N/A

**Chemicals, peptides, and recombinant proteins**		

Ampuwa water (refered to as dH_2_O)	Fresenius Kabi	Cat# 06605508
Exonuclease I, high concentration	Applied Biosystems	Cat# 720735KU
FastAP thermosensitive alkaline phosphatase	Applied Biosystems	Cat# EF0651
BamHI-HF restriction enzyme	New England Biolabs	Cat# R3136S
XhoI restriction enzyme	New England Biolabs	Cat# R0146S
Loading dye, purple (6×)	New England Biolabs	Cat# B7024S
T4 DNA ligase	New England Biolabs	Cat# M0202S
FuGENE 6 transfection reagent	Promega	Cat# E2691
MangoTaq DNA polymerase	Meridian (Bioline)	Cat# BIO-21083-BL
100 mM dNTP set	Thermo Fisher Scientific	Cat# 10083252
Deoxynucleotide (dNTP) Solution Mix (10 mM each dNTP)	New England Biolabs	Cat# N0447S
Gibco RPMI 1640 medium	Thermo Fisher Scientific	Cat# 21875034
Gibco Dulbecco’s modified Eagle’s medium (DMEM), high glucose, pyruvate	Thermo Fisher Scientific	Cat# 41966052
Gibco Dulbecco’s phosphate-buffered saline (DPBS), without calcium, without magnesium	Thermo Fisher Scientific	Cat# 14190144
Gibco Trypsin-EDTA (0.25%), phenol red	Thermo Fisher Scientific	Cat# 25200056
Gibco penicillin-streptomycin (10,000 U/mL)	Thermo Fisher Scientific	Cat# 11548876
Fetal bovine serum (FBS) superior	Sigma-Aldrich	Cat# S0615
Amphotericin B	Sigma-Aldrich	Cat# A2942

**Critical commercial assays**		

Q5 high-fidelity DNA polymerase kit	New England Biolabs	Cat# M0491S
QIAGEN multiplex PCR kit	QIAGEN	Cat# 206143
Q5 site-directed mutagenesis kit	New England Biolabs	Cat# E0554S
GenElute PCR clean-up kit	Sigma-Aldrich	Cat# NA1020
GenElute plasmid midiprep kit	Sigma-Aldrich	Cat# PLD35
GenElute plasmid miniprep kit	Sigma-Aldrich	Cat# PLN70
miRNeasy mini kit	QIAGEN	Cat# 217004
High-capacity cDNA reverse transcription kit	Applied Biosystems	Cat# 4368814
Zero Blunt TOPO PCR cloning kit for sequencing	Thermo Fisher Scientific	Cat# 450031

**Deposited data**		

pET01 vector sequence	This paper (full plasmid sequencing) MoBiTec GmbH, Gottingen, Germany	Document S1 (Data S1)
Data of *OTOF* variants generated using this method	This paper	Document S1 (Figures S1–S4)

**Experimental models: Cell lines**		

Human: HCT116 cell line	ATCC, Manassas, VA, USA	https://www.atcc.org/products/ccl-247
Human: HEK293T cell line	ATCC, Manassas, VA, USA	https://www.atcc.org/products/crl-3216

**Oligonucleotides**		

PCR primer 02 F: GATGGATCCGCTTCCTGCCCC, with and without 5′ 6-carboxyfluorescein (FAM) label	MobiTec GmbH^4^	https://www.mobitec.com/media/datasheets/mobitecgmbh/Exontrap-Handbook.pdf
PCR primer 03 R: CTCCCGGGCCACCTCCAGTGCC	MobiTec GmbH^4^	https://www.mobitec.com/media/datasheets/mobitecgmbh/Exontrap-Handbook.pdf
cDNA primer 01: GATCCACGATGC	MobiTec GmbH^4^	https://www.mobitec.com/media/datasheets/mobitecgmbh/Exontrap-Handbook.pdf
M13 primer F: GTAAAACGACGGCCAG	N/A	N/A
M13 primer R: CAGGAAACAGCTATGACC	N/A	N/A
Self-designed *OTOF* primers	This paper	Document S1 (Table S1)

**Recombinant DNA**		

Plasmid: Exontrap vector pET01	MoBiTec GmbH	Cat# PET01

**Software and algorithms**		

ApE, A plasmid Editor	Davis and Jorgensen^5^	https://jorgensen.biology.utah.edu/wayned/ape/
Alamut Visual Plus	Sophia Genetics	https://www.sophiagenetics.com/sophia-ddm-for-genomics/alamut-visual-plus/
Ensembl	Harrison et al.^6^	https://www.ensembl.org/index.html
UCSC Genome Browser	Raney et al.^7^	https://genome.ucsc.edu/cgi-bin/hgBlat
Primer3	Untergasser et al.^8^	https://primer3.ut.ee/
NEBaseChanger	New England Biolabs	https://nebasechanger.neb.com/
T_m_ calculator	New England Biolabs	https://tmcalculator.neb.com/#!/main
T_m_ for Oligos Calculator	Promega	https://www.promega.de/resources/tools/biomath/tm-calculator/
SNPCheck	EMQN and Certus Technology	https://genetools.org/SNPCheck/snpcheck.htm
GeneMapper Software 5	Applied Biosystems	https://www.thermofisher.com/order/catalog/product/4370784

**Other**		

Arium Pro ultrapure water system (refered to as H_2_O)	Sartorius	Cat# H2OPRO-UV-T
Heracell 150 CO_2_ incubator	Thermo Fisher Scientific	N/A
6-well CytoOne plate, TC-treated	Starlab	Cat# CC7682-7506
Screw cap tube, 50 mL	SARSTEDT	Cat# 62.547.254
SafeSeal reaction tube, 1.5 mL, PP, PCR performance tested (referred to as Eppi in this protocol)	SARSTEDT	Cat# 72.706,400
PCR SingleCap 8-strip tubes, 0.2 mL	Biozym	Cat# 710988
Sterile petri dishes, 94 × 16 mm	Greiner	Cat# 633181
Parafilm M sealing film	Merck	Cat# HS234526B
Fresco 21 microcentrifuge	Thermo Fisher Scientific	Cat# 75002425
S1000 thermal cycler with dual 48/48 fast reaction module	Bio-Rad	Cat# 1852148
Gel documentation system	NIPPON Genetics EUROPE	N/A
NanoDrop One^C^	Thermo Fisher Scientific	Cat# ND-ONEC-W
Thermomixer comfort heat block	Eppendorf	Cat# 5355 000.011
3730xl DNA analyzer	Applied Biosystems	Cat#A41046

## Materials and equipment

**Table:** Supplemented RPMI/DMEM medium

Reagent	Final concentration	Amount
RPMI 1640 medium **or** Dulbecco’s Modified Eagle medium, high glucose, pyruvate	–	500 mL
Penicillin-Streptomycin (10,000 U/mL)	∼90 U/mL	5 mL
FBS Superior	∼9%	50 mL
Amphotericin B (250 μg/mL)	∼700 ng/mL	1.6 mL
**Total**	**N/A**	**556.6 mL**

***Note:*** Store at 4°C for up to 1 month and pre-warm approximately 30 min (dependent on water bath) to 37°C before usage.

**Table:** LB agar plates with selection marker (50 μg/mL)

Reagent	Amount
Tryptone/Peptone ex casein pancreatically digested	3 g
Sodium chloride	3 g
Yeast extract	1.5 g
Agar-Agar, Kobe I	4.5 g
Ampicillin (50 mg/mL) **or** kanamycin (10 mg/mL)	300 μL **or** 1.5 mL
H_2_O	300 mL
**Total**	**≈ 300 mL**

***Note:*** Autoclave the LB agar mixture and let it cool down to ∼60°C before adding the antibiotics. Then pour into 94 x 16 mm petri dishes and let them solidify. Store in a dark place inverted in a sealed plastic bag to prevent condensation and drying at 4°C for up to 2 weeks.

## Step-by-step method details

### Amplify your region of interest

**Timing:2–3 h**

1. Determine PCR conditions.
  ***Note:*** The amplification of genomic DNA regions of interest can be performed using other PCR kits, provided that the DNA polymerase used possesses proofreading activity. In our protocol, we used the Q5 High-Fidelity DNA Polymerase Kit by NEB.
  a. Calculate the annealing temperature of your designed primers according to the manufacturer’s instructions.
    ***Note:*** We use the NEB T_m_ Calculator website (https://tmcalculator.neb.com/#!/main) recommended for the Q5 High-Fidelity DNA Polymerase Kit with the primer concentration set to 500 nM.
  b. Calculate the extension time of the PCR according to the manufacturer. We use 25 s per kilobase of amplicon.
2. Perform the PCR according to the manufacturer with your previously designed primers (https://doi.org/10.17504/protocols.io.be6bjhan).
  a. Include a negative control with dH_2_O instead of DNA.

**Table:** Q5 High-Fidelity PCR reaction master mix

Reagent	Volume (one sample)
5× Q5 Reaction Buffer	5 μl
10 mM dNTPs	0.5 μl
10 μM Primer F	1.25 μl
10 μM Primer R	1.25 μl
dH_2_O	15.75 μl
Q5 High-Fidelity Polymerase	0.25 μl
Total volume	24 μL (add 1 μL of genomic healthy control DNA)

**Table:** PCR cycling conditions

Steps	Temperature	Time	Cycles
Initial denaturation	98°C	30 s	1
Denaturation	98°C	10 s	35 cycles
Annealing	T_m_	20 s	
Extension	72°C	25 s/kb of amplicon	
Final extension	72°C	2 min	1
Hold	4°C	forever

3. While the PCR is running, prepare a 1.0–1.5% agarose gel.
4. Mix 5 μl of PCR product with 1 μl of 6× Loading Dye. Load the agarose gel with this sample. Run at 120 V for at least 30 min.
5. If the band on the agarose gel shows the correct amplicon size with a strong intensity, repeat the PCR with a larger set-up.

***Note:*** We usually perform a four times set-up (repeating exactly as above for four identical reactions). Retain and combine the PCR volume of each reaction together in a standard autoclaved 1.5 mL Eppendorf (Eppi) tube for step 6.

***Note:*** Refer to our troubleshooting for this step, if multiple bands occur, see troubleshooting.

6. Clean-up PCR products with a standard PCR clean-up kit.

***Note:*** We use the GenElute PCR Clean-Up Kit (Sigma Aldrich) according to the manufacturer’s instruction (https://www.sigmaaldrich.com/deepweb/assets/sigmaaldrich/product/documents/786/801/na1020bul.pdf). We recommend eluting with 50 μl of dH_2_O water instead of the elution buffer of the supplied kit.

### Digest PCR product and vector with restriction enzymes

**Timing:2 h**

Digesting the PCR product and vector with restriction enzymes will create sticky ends for ligation of the amplicon into pET01.

7. Determine the optimum buffer and temperature for the restriction enzymes according to the manufacturer’s instructions (https://www.neb.com/en/tools-and-resources/usage-guidelines/nebuffer-performance-chart-with-restriction-enzymes).

***Note:*** The example below assumes the same enzyme-dependent incubation temperature and buffer is suitable for a double digestion. If different restriction enzymes are used, ensure that they are compatible for a double digestion. Otherwise, perform one digestion at a time according to the respective manufacturer’s protocol.

8. Prepare the digestion mixture in a 1.5 mL Eppi:

**Table:** Digestion mixture

Reagent	Volume (one sample)
PCR product or pET01 plasmid	50 μl
10× rCutSmart Buffer	10 μl
dH_2_O	36 μl
Restriction enzyme 1 (New England Biolabs)	2 μl
Restriction enzyme 2 (New England Biolabs)	2 μl
Total volume	100 μl

9. Incubate the digestion mixture on a heat block at the enzyme-dependent temperature for 1 h.
10. Clean-up again with the GenElute PCR Clean-Up Kit (Sigma Aldrich), as used in step 6, also eluting with 50 μl of water.
11. Load a 1.0–1.5% agarose gel with 1 μl of the respective digestion mixture, pre-mixed with 4 μl of dH_2_O and 1 μl of 6× Loading Dye.

**CRITICAL:** Ensure that the vector is fully digested, as indicated by one band at around 4.2 kb.

**Pause point:** After the clean-up procedure, the PCR product can be stored at −20°C or ligation can be performed immediately after.

***Note:*** The digested vector can also be stored at −20°C for later use. As only 1 μl of digested vector is needed for ligation, this can be used for creating more than one pET01 construct.

### Ligate your PCR product into pET01 and perform transformation

**Timing:2.5 h + incubation for 16–24 h**

After ligating the respective insert into the digested pET01 vector, the ligation product is transformed into competent *E. coli* cells. Following transformation, the bacteria are plated on LB agar containing the antibiotic ampicillin for selection. Several resulting colonies can then be picked and screened by colony PCR followed by sequencing to confirm the presence and correctness of the insert prior to use in downstream applications such as site-directed mutagenesis and transfection.

12. Prepare the ligation mixture in a 1.5 mL Eppi:

**Table:** Ligation mixture

Reagent	Volume (one sample)
Digested PCR product	3 μl
Digested pET01 vector	1 μl
dH_2_O	13.5 μl
10× buffer for T4 DNA Ligase	2 μl
T4 DNA Ligase	0.5 μl
Total volume	20 μl

13. Incubate 10 min at 19°C–25°C.

**Pause point:** The ligation can also be incubated longer at 19°C–25°C for up to 16 h or can be stored at −20°C and transformed later following the 10 min incubation.

14. Transform into competent *E.coli* and incubate 16–24 h. We use the following protocol: New England Biolabs: High Efficiency Transformation Protocol (C2987H).^9^ https://www.protocols.io/view/high-efficiency-transformation-protocol-c2987h-95qpvoqxl4o1/v3.
  a. Warm the bottle of SOC medium to 19°C–25°C, put prepared selection plates (LB agar plates with ampicillin) into an incubator to warm to 37°C.
  b. Thaw one tube of C2987H NEB 5-alpha competent cells on ice for 10 min.
  c. Add 5 μl of the ligation mixture onto the cells and carefully flick the tube four to five times to mix.
    **CRITICAL:** Do not vortex.
  d. Incubate on ice for 30 min. Preheat a heat block to 42°C.
    ***Note:*** If you anticipate that the first heat block may be slow to cool down, preheat a second heat block to 37°C.
  e. Heat shock on the heat block for exactly 30 s.
  f. Place on ice for 5 min. Adjust heat block to 37°C.
  g. Pipette 950 μl of SOC medium onto the cells.
  h. Place the sample onto the heat block for 1 h at 37°C, while shaking (250–300 rpm).
  i. Mix the cells thoroughly by flicking the tube and inverting.
  j. Dilute the cells in SOC medium. A ∼1:10 dilution usually works (225 μl of SOC medium and 25 μl of cells).
    ***Note:*** Keep the rest of the cells stored at 4°C, in case no colonies are observed the next day. Then plate the cells out undiluted. If colonies are observed, freeze the cells with 1:1 40% glycerol at −80°C as a backup glycerol stock.
  k. Spread the diluted cells onto the LB agar selection plates (with ampicillin) and place in incubator at 37°C for 16–24 h.
    **Pause point:** The next day, either proceed with the following steps or seal the LB agar plate with parafilm to avoid dehydration, storing it at 4°C for future processing.

### Colony PCR to identify a pET01 clone with insert

**Timing:2–4 h**

On the LB agar plates containing ampicillin, only *E.coli* clones with the transformed vector should grow. Using a colony screening method, we select a clone that contains the vector with the insert and confirm its identity via PCR, assessing the correct insert size. Subsequent Sanger sequencing will validate this clone.

15. Prepare for the colony PCR.
  a. Label a pre-warmed (37°C) new LB agar plate containing ampicillin with a grid (called grid plate) and sample name.
  b. Calculate the PCR extension time using the formula: 40 s∗(0.430 kb + the kilobase pair length of your insert) = extension time in seconds.
  c. Calculate the annealing temperature of the primers using the Promega T_m_ Calculator (https://www.promega.de/resources/tools/biomath/tm-calculator/) setting the primer concentration to 300 nM. Use the average of the two calculated temperatures.
16. Prepare a Master Mix depending on the number of colonies you intend to screen.

***Note:*** We usually screen five to ten individual colonies. Include a negative control with no colony material added.

**Table:** Colony screen Master Mix

Reagent	Volume (one sample)
5× Reaction Buffer, colored	2.5 μl
MgCl_2_ (50 mM)	0.5 μl
dNTPs (100 mM)	0.125 μl
10 μM Primer 02 F	0.375 μl
10 μM Self-designed insert primer (reverse)	0.375 μl
dH_2_O	8 μl
MangoTaq DNA polymerase	0.125 μl
Total volume	12 μl

17. Divide the colony screen Master Mix into PCR tubes.
  a. Using a sterile micropipette tip, pick one colony from the plate and place the tip onto a square of your grid plate.
  b. Insert this tip into a PCR tube containing the Master Mix.
  c. Incubate the tip for at least one minute.
  d. Re-attach the pipette and pipette up and down to ensure thorough mixing before discarding the tip.
  e. Repeat this process for the desired number of colonies to be screened.
18. Incubate the grid plate at 37°C for 16–24 h.
19. Run the PCR using the following cycling conditions. Concurrently, prepare a 1.0–1.5% agarose gel.

**Table:** PCR cycling conditions

Steps	Temperature	Time	Cycles
Initial denaturation	95°C	5 min	1
Denaturation	95°C	30 s	35 cycles
Annealing	T_m_	30 s	
Extension	72°C	40 s/kb of amplicon	
Final extension	72°C	5 min	1
Hold	4°C	forever

20. Load 5 μl of the PCR product mixed with 1 μl of 6× Loading Dye onto the 1.0–1.5% agarose gel.

**CRITICAL:** Since the forward primer anneals to vector-specific sequence and the reverse primer anneals to insert-specific sequence, a band should only appear on the agarose gel for colonies containing the vector with insert. The expected band size will be the insert length plus approximately 430 bp, depending on the restriction enzymes used. Absence of a band indicates that the colony does not contain the insert.

21. Select colonies for Sanger sequencing based on gel results, typically choosing three colonies that display a band at the expected size.

***Note:*** To conserve resources, consider sequencing the remaining PCR product with standard overnight Sanger sequencing, followed by preparing a single 16 h culture after receiving and analyzing the sequencing results. For expedited processing, you may prepare 16 h (LB-amp) cultures for all colonies showing the correct band, wait for sequencing the next day and perform plasmid isolation only on the Sanger-confirmed 16 h culture.

22. Prepare the samples for sequencing with a standard PCR clean-up reaction.

***Note:*** The following shows an example of how this is done but should be adjusted to Sanger sequencing sample requirements in each lab.

**Table:** PCR clean-up Master Mix

Reagent	Volume (one sample)
dH_2_O	3.625 μl
Exonuclease I	0.075 μl
FastAP	0.3
Total volume	4 μl

23. Mix 6 μl of the colony screen PCR product with 4 μl of the PCR clean-up Master Mix.
24. Run the following PCR program:

**Table:** PCR clean-up cycling conditions

Temperature	Time
37°C	15 min
85°C	15 min
4°C	hold

25. Prepare a Master Mix for sequencing, which includes the primer for sequencing.

**Table:** Sanger sequencing Master Mix

Reagent	Volume (one sample)
dH_2_O	9 μl
PCR primer 02 F	3 μl
Total volume	12 μl

***Note:*** We usually just use the vector primer PCR primer 02 F for sequencing. If the insert is bigger than 600 base pairs, we also prepare another sample to sequence with the self-designed insert primer (reverse).

26. Add 3 μl of the PCR clean up product with one sample of the Sanger sequencing Master Mix into tubes appropriate for your sequencing facility.
27. Confirm the correct insert with analysis of Sanger sequencing results.

### 16 h culture and plasmid isolation

**Timing:10 min + 16 h incubation + 1.5 h plasmid isolation**

After confirming a clone with the correct insert with Sanger sequencing, set up a 16 h culture.

**CRITICAL:** Compared to other vectors used in our lab, we have observed lower pET01 vector concentration yields when using standard Miniprep kits for plasmid isolation. To avoid the need for repeated plasmid isolations, particularly in cases where repeat transfections may be needed, duplicating/triplicating transfections or transfecting the same construct in multiple cell lines, we advise using a Midiprep kit (e.g. GenElute Plasmid Midiprep Kit (Sigma-Aldrich)) for higher yield.

28. Aliquot 20 mL of LB medium into a tube suitable for 16 h incubation, and add 10.5 μl of ampicillin (stock concentration=50 mg/mL, final concentration=25 μg/mL).
29. Using a pipette tip, inoculate the Sanger confirmed colony from the grid plate into the LB medium with ampicillin.

**Pause point:** Incubate the culture for 16 h at 37°C, while shaking at 150–400 rpm in an incubator.

***Note:*** After incubation, mix 500 μl of the 16 h culture with 500 μl of 40% glycerol in a vial suitable for freezing and store at −80°C for back-up. Process the remaining sample in step 30.

30. Follow the manufacturer’s instructions of the Midiprep kit for plasmid isolation (https://www.sigmaaldrich.com/deepweb/assets/sigmaaldrich/product/documents/605/823/pld35bul.pdf?srsltid=AfmBOoptscaVUPIPzd3Aflc9oHWn56qB5vyPXjj90raeOHjrIIGAC76q) with following modification:
  a. For a more concentrated eluate, use 500 μl of preheated (65°C) dH_2_O as the elution solution and incubate on the column for 10 min before elution.
31. Measure the concentration of your isolated plasmid using a spectrophotometer.
32. Validate the plasmid with Sanger sequencing at your local sequencing facility.

### Create a variant minigene via site-directed mutagenesis

**Timing:3–4 days**

After isolating the wild-type construct, the following PCR-based mutagenesis method allows for simple introduction of specific variants using pre-designed mutagenesis primers. This technique can facilitate insertions, deletions or single-base pair exchanges. We follow the protocol available at https://www.protocols.io/view/q5-site-directed-mutagenesis-e0554-8n92ldqov5br/v2^10^ and listed in the following.

33. If not already previously done, measure the concentration of the wild-type template plasmid.
34. Dilute the plasmid to a concentration of 20 ng/μl using dH_2_O in a total volume of 15 μl.
35. Prepare the following Master Mix with standard components supplied in the Q5 Site-Directed Mutagenesis Kit:

**Table:** Q5 SDM Master Mix

Reagent	Volume (one sample)
Q5 Hot Start High-Fidelity 2× Master Mix	12.5 μl
10 μM Mutagenesis Primer (forward)	1.25 μl
10 μM Mutagenesis Primer (reverse)	1.25 μl
Wild-type plasmid (20 ng/μl)	1 μl
dH_2_O	9 μl
Total volume	25 μl

36. Mix by pipetting up and down and spin down shortly in a table centrifuge.
37. Calculate the annealing temperature and the extension time of your PCR.

***Note:*** We use the annealing temperature provided by the NEBaseChanger website (https://nebasechanger.neb.com/).

38. Run the following PCR program according to the manufacturer protocol:

**Table:** PCR cycling conditions

Steps	Temperature	Time	Cycles
Initial denaturation	98°C	30 s	1
Denaturation	98°C	10 s	25 cycles
Annealing	T_m_	10 s	
Extension	72°C	20 s/kb of plasmid	
Final extension	72°C	2 min	1
Hold	4°C	forever

39. Prepare a transformation by performing step 14a.
40. Thaw a tube of NEB 5-alpha competent *E. coli* on ice for 10 min.
41. Assemble the following reagents in a 1.5 ml Eppendorf tube, then gently pipette up and down.

**Table:** KLD enzyme Mix

Reagent	Volume (one sample)
PCR Product	1 μl
2× Reaction Buffer	5 μl
10× KLD Enzyme Mix	1 μl
dH_2_O	3 μl
Total volume	10 μl

42. Incubate the KLD enzyme reaction mixture at 19°C–25°C for 5 min, while allowing *E. coli* cells to thaw on ice for the same period (around 10 min total incubation time).
43. Perform transformation starting with step 14c by adding 5 μl of the KLD enzyme-treated mixture.

**Pause point:** Incubate the plate for 16–24 h at 37°C. The next day, either proceed with the following steps or seal the LB agar plate with parafilm to avoid dehydration, storing it at 4°C for future processing.

44. Screen the colonies for the desired insert, following the same procedure from steps 15–27.
45. After validating a clone containing the correct insert and variant, set up a 16 h culture for plasmid isolation the next day (same as steps 28–31).

### Transfecting minigenes

**Timing:30 min (day 1) + 30 min (day 2)**

Transfect wild-type and variant plasmid constructs into the desired cell line to utilize the inherent transcription and splicing processes of the cells. Optimize the transfection procedure for your cell line of choice. This protocol describes transient transfection in HCT116 cells with RPMI medium. Results were replicated in HEK293T cells, using DMEM medium instead.

**CRITICAL:** Seed 4 × 10^5^ cells per well in a 6-well plate the day before transfection, ensuring 60–70% confluence on the day of transfection.

***Note:*** For transfection, include not only the wild-type and variant construct, but also an empty pET01 vector control and a transfection negative control.

46. Prewarm FBS-free RPMI medium to 37°C.
47. In a 1.5 ml Eppi, dilute 1 μg of each isolated plasmid construct with FBS-free RPMI medium to a total volume of 95 μl.
48. Add 3 μl of FuGene 6 Transfection Reagent to each transfection mix. Gently mix.
49. Incubate at 19°C–25°C for 15 min.
50. During incubation, change the medium in the 6-well plate to 2 mL of pre-warmed FBS-free RPMI medium per well.
51. Slowly add the transfection mix dropwise to the respective well using a pipette.
52. Gently mix the plate by tilting.
53. Incubate at 37°C humidified incubator with 95% humidity and 5% CO_2_ for 24 h.
54. 24 h post-transfection change the medium back to supplemented medium (with FBS).
55. Incubate under the same conditions for another 24 h.

### Isolate RNA and synthesize cDNA

**Timing:3–4 h + 2.5 h incubation**

**Timing: Variable (for steps 65–69)**

48 h post-transfection, isolate RNA from your transfected cells followed by two-step RT-PCR. For the first step use a primer with sequence provided by the manufacturer that anneals to the pET01 vector sequence (step 57). For the second step, use primers amplifying across exons A and B (step 61).

56. Isolate total RNA 48 h post-transfection using a standard RNA isolation kit. We recommend QIAGEN’s miRNeasy Mini Kit and protocol (https://www.qiagen.com/us/resources/resourcedetail?id=da6c8d17-58c4-411c-a334-bc1754876db3&lang=en) with the following modifications:
  **CRITICAL:** Pre-chill centrifuge to 4°C and pre-warm RNase free water to 55°C.
  a. Remove the medium from the well plate and add 2 ml of DPBS in each well.
  b. Detach the cells by pipetting and transfer to 2 mL Eppi.
  c. Pellet the cells by centrifugation at 3,000 g and 4°C for 5 min.
  d. Discard the supernatant and flick the Eppi to dissolve the pellet in the residual DPBS.
  e. Add 700 μl of QIAzol Lysis Reagent and vortex for 1 min.
  f. Incubate at 19°C–25°C for 5 min.
  g. Add 140 μl of chloroform and shake vigorously for 15 s.
  h. Incubate for 5 min at 19–25°C.
  i. Centrifuge for 15 min at 12,000 g at 4°C.
    **CRITICAL:** Ensure the formation of three phases: A pink organic phase (bottom), a white interphase and an aqueous top phase containing the RNA. If the phases are unclear, re-centrifuge or check if all components were added.
  j. Transfer 350 μl of the upper phase to a new Eppi and mix with 525 μl nuclease free 100% ethanol.
    **CRITICAL:** Do not transfer the white interphase or pink organic phase. If this proves difficult, transfer a smaller volume of the upper phase at a time until reaching the 350 μl volume or re-centrifuge ensure separation of the phases.
    ***Note:*** From now on warm the centrifuge to 19°C–25°C again or use another centrifuge at room temperature.
  k. Transfer 700 μl of the mixture to the provided column and centrifuge for 15 s at 10,000 g at 19°C–25°C.
  l. Decant the flow-through.
  m. Add the remaining mixture from step j to the column and centrifuge for 15 s at 10,000 g (19°C–25°C) and decant the flow-through.
  n. Add 700 μl of RWT buffer to the column. Centrifuge for 15 s at 10,000 g (19°C–25°C) and decant the flow-through.
  o. Add 500 μl of the RPE buffer to the column. Centrifuge for 15 s at 10,000 g (19°C–25°C) and decant the flow-through.
  p. Repeat step o.
  q. Transfer the column to a new collection tube and centrifuge for 1 min at full speed (19°C–25°C).
  r. Transfer the column to a new Eppi and add 35 μl of the pre-warmed RNase free water directly onto the center of the column.
  s. Incubate for 1 min.
  t. Centrifuge for 1 min at 10,000 g (19°C–25°C) to elute the RNA.
    **CRITICAL:** Always keep the isolated RNA on ice from this point forward.
57. Measure the RNA concentration with a spectrophotometer.

**CRITICAL:** Ensure RNase free water is used as the blank. See troubleshooting.

**Pause point:** Store the RNA at −80°C, or proceed immediately with step 58. When continuing directly with step 58, store any remaining RNA at −80°C.

58. Dilute the RNA in RNase free water to 2 μg in a volume of 10 μl.
59. Prepare a RT-PCR Master Mix with the High-Capacity cDNA Reverse Transcription Kit (Applied Biosystems) and the cDNA primer 01 on ice:

**Table:** RT-PCR Master Mix

Reagent	Volume (one sample)
10× RT Buffer	2 μl
25× dNTP Mix (100 mM)	0.8 μl
cDNA Primer 01 (100 μM)	2 μl
MultiScribe Reverse Transcriptase	1.0 μl
RNase free H_2_O	4.2 μl
Total volume	10 μl

60. Gently mix and aliquot in individual PCR tubes.
61. Pipette 10 μl of RNA (2 μg) into the PCR tube, gently mix by pipetting.
62. Run the PCR with following program:

**Table:** PCR cycling conditions

Temperature	Time
25°C	10 min
37°C	120 min
85°C	5 min
4°C	hold

**Pause point:** Store labeled cDNA at −20°C until ready to perform the final PCR with PCR primer 02 F and 03 R. This will amplify the region between Exon A and Exon B of the pET01 vector.

63. Prepare a Master Mix for the final PCR with a standard PCR kit.

**CRITICAL:** If fragment analysis is required to quantify different amplicons, use one FAM-labeled primer for this PCR (e.g. FAM-labeled forward primer and a non-FAM-labeled reverse primer). Additionally, to prevent interference of FAM-labeled amplicons with sequencing, run a parallel PCR with non-FAM-labeled primers. Be sure to include controls.

***Note:*** We obtained good PCR results using the QIAGEN Multiplex PCR Kit. However, depending on the sample, other PCR kits might be more suitable. Refer to our troubleshooting guide if problems occur, see troubleshooting.

**Table:** Final PCR Master Mix

Reagent	Volume (one sample)
2× QIAGEN Multiplex PCR Master Mix	5 μl
10 μM (FAM-)PCR primer 02 F	1 μl
10 μM PCR primer 03 R	1 μl
dH_2_O	11.5 μl
Add cDNA	1.5 μl
Total volume	20 μl

64. Run a touchdown PCR program. Make adjustments if needed.

**Table:** PCR cycling conditions

Steps	Temperature	Time	Cycles
Initial denaturation	95°C	5 min.	1
Denaturation	95°C	30 s	4 cycles
Annealing	62°C	30 s	
Extension	72°C	30 s	
Denaturation	95°C	30 s	6 cycles
Annealing	59°C	30 s	
Extension	72°C	30 s	
Denaturation	95°C	30 s	31 cycles
Annealing	56°C	30 s	
Extension	72°C	30 s	
Final extension	72°C	8 min	1
Hold	4°C	forever

65. Prepare a 1.0–1.5% agarose gel and run the PCR products with 6× Loading Dye for at least 1 h at 120 V. See expected outcomes for an example gel.

***Optional:*** Cloning to isolate single sequences in instances where splicing yields multiple amplicons.

If multiple bands are observed on the final agarose gel, direct sequencing of the RT-PCR product may result in noisy or unusable sequencing results. To avoid this, you can either clone the RT-PCR products into another vector or perform gel extraction.

***Note:*** We have achieved good results with the QIAquick Gel Extraction Kit (QIAGEN). However, if the splice products differ by only a very small number of base pairs, cloning the final PCR products into a vector is preferable for cleaner sequencing results. For instructions on subsequent cloning of the RT-PCR products into the pCR4Blunt TOPO vector (Fisher Scientific), follow the next steps.

**CRITICAL:** Ensure that the RT-PCR products used in these steps are generated with non-FAM labeled primers. Use 30-60 μl of PCR product (depending on its intensity on the agarose gel) and purify it using a standard PCR clean-up column kit. Elute the product in 30 μl of dH_2_O.

66. Ligate the RT-PCR product into the pCR4Blunt TOPO vector according to the manufacturer (https://assets.thermofisher.com/TFS-Assets/LSG/manuals/topobluntseq_man.pdf):

**Table:** Ligation mixture

Reagent	Volume (one sample)
PCR product	4 μl
Salt solution	1 μl
pCR4Blunt TOPO vector	1 μl
Total volume	6 μl

67. Gently mix the ligation reaction, briefly spin down in a tabletop centrifuge and incubate for 15–30 min at 19°C–25°C.
68. Transform all 6 μl into NEB 5-alpha Competent *E.coli* described in step 14. This time, plate out 250 μl of undiluted transformed bacteria onto LB agar plates (with kanamycin).

**Pause point:** Incubate 16–24 h at 37°C.

69. Perform a colony PCR and send the PCR products for Sanger sequencing, as described in steps 15–27. This time use M13 forward and reverse primers.

### Quantification

Fragment Analysis is used to quantify different sizes of PCR products labeled with fluorescent dye (FAM label) using capillary electrophoresis. It is especially useful to detect small quantities of splice products that cannot be visualized on an agarose gel. Furthermore, fragment analysis by capillary array electrophoresis achieves high sensitivity to allow for relative quantification of multiple bands and it is especially helpful for providing high resolution to detect base pair differences of 1 bp or more that may not be easily resolved on agarose gels.

70. Prepare FAM-labeled final PCR products as in steps 57–63 for Fragment Analysis.
71. Dilute samples with suitable buffer according to internal lab standard.
72. Mix samples with “orange dye” labeled size standard according to internal lab standard.
73. Denature samples at 96°C for 4 min.
74. Load samples onto the fragment analyzer. We used the ABI3730 XL DNA analyzer with the following conditions: injection voltage of 1.6 V and injection time of 15 s, run voltage of 15 kV and run time of 2500 s.
75. Analyze data with fragment analysis software such as GeneMapper 5 (Applied Biosystems).

***Note:*** Refer to our troubleshooting.

## Expected outcomes

In this protocol, we used the pET01 vector to study the splicing effects of inherited patient variants in *OTOF*. Variants can affect pre-mRNA splicing through various mechanisms, leading to different consequences. Disruptions to the highly conserved 5′ splice donor, 3′ splice acceptor sites or splicing regulatory elements (SREs) can interfere with the recognition by trans-acting splicing factors. Additionally, some variants can also activate cryptic splice sites. The consequences of these disruptions can include exon skipping, partial or complete intron retention or exon truncation. These events can result in insertions or deletions, which may preserve the reading frame or disrupt it, potentially introducing a premature stop codon, typically resulting in a truncated and often non-functional protein.^1^^,^^11^

**Figure 2:**
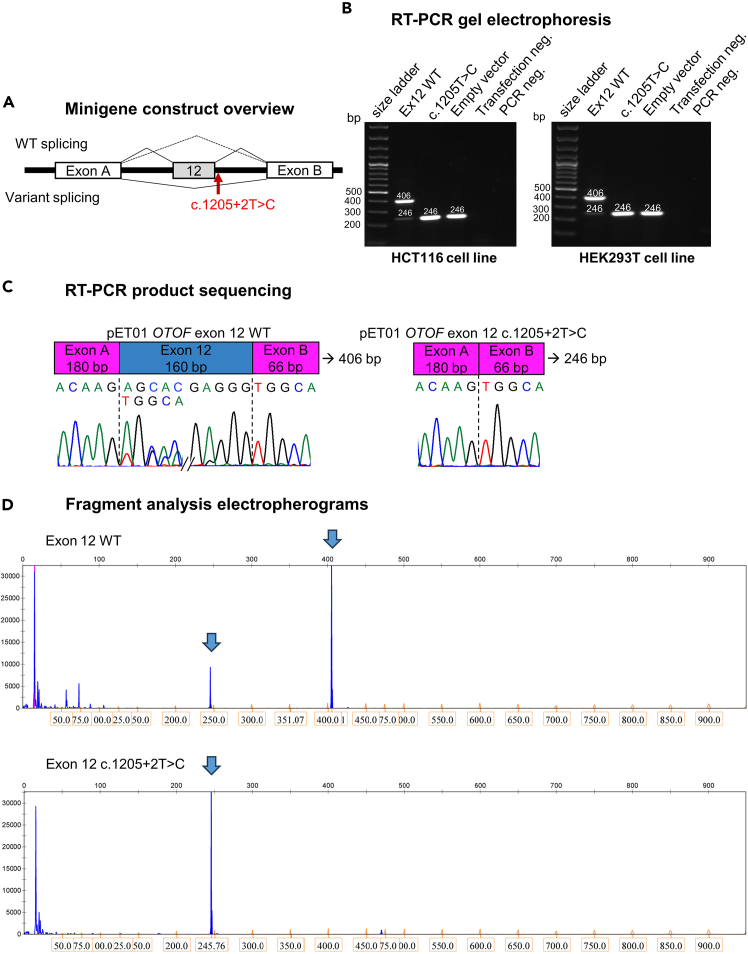
Minigene assay of the c.1205+2T>C *OTOF* variant (A) Minigene construct schematic with Exon 12 of *OTOF* cloned in between exon A and B of the vector. The red arrow displays the position of the variant. The WT and variant construct splicing pattern is indicated on top and bottom, respectively. (B) Visualization of final RT-PCR products after gel electrophoresis. Minigenes were transfected into HCT116 (left) and HEK293T (right) cells. (C) Direct Sanger sequencing results of RT-PCR products of HCT116 cells. The primer PCR 02 F, aligning to exon A of pET01 was used as a primer for sequencing. Exon A and B of the vector are indicated by pink boxes, exon 12 by a blue box. The wild-type construct sample shows normal exon 12 splicing and skipping of exon 12. The variant sample shows exon 12 skipping only. (D) Electropherogram of one triplicate of the final RT-PCR products from HCT116 cells. The blue arrows point to peaks that correspond to the sizes indicated on the agarose gel in panel (B).

When analyzing splicing patterns in the minigene model, it is essential to compare results to the wild-type minigene, which should express the correctly spliced exons. Figure 2 illustrates exon skipping caused by a variant in *OTOF.* Examples of variants assayed with this protocol and results shown in two cell lines are accessible in the supplementary material (Figures S1–S4).

## Limitations

While the minigene assay is a widely used, robust, quick and cost-effective method for assessing splice effects of genetic variants, providing validation of splice effects on the RNA-level, it has certain limitations.^12^^,^^13^

The insert for the minigene is only a small fragment of the full-length gene, which can be an advantage to reduce complexity and focus on the most important regions. However, this simplification can also be a drawback, as the absence of the full genomic context may cause regulatory elements located far from the splice sites to be missed, which can influence splicing.

Another potential limitation is the choice of cell line for transfection, as it may affect splicing outcomes. Ideally, if available, a cell line that closely mimics the tissue-specific splicing of the gene of interest should be used to potentially improve the relevance of the results.

In addition, this assay requires cloning, transfection and subsequent analysis, which can be labor and resource intensive.

Furthermore, while analysis of mRNA in this assay provides valuable insight into changes in splice patterns, interpreting the subsequent protein presence and function should be evaluated with caution due to many factors influencing translation and post-translational modifications.

Some mis-splicing events, such as exon skipping, have been observed even when expressing the wild-type minigene, likely due to the loss of regulatory sequences within the intronic regions included in the construct or the strong artificial splice sites of the vector. This limitation, combined with the size restrictions of the cloned DNA segments, makes minigene assays potentially unsuitable for functionally assessing deep intronic variants in very large introns. Therefore, each variant requires careful evaluation to determine the most appropriate functional assay to use.

While our experimental findings highlight some limitations in minigene-based splicing analysis, we have validated the use of a customizable minigene assay within clinical investigations and successfully identified variants causing aberrant splicing. In the absence of appropriate patient samples for RNA investigation, this is a critical tool for clinical variant investigation.

## Troubleshooting

### Problem 1

The first PCR product presents as multiple bands on the agarose gel (major step 1).

### Potential solution

• Perform a gradient PCR with increased T_m_.
• Choose a different DNA sample (if available) to avoid SNPs.
• Design new primers.

### Problem 2

The final RT-PCR product shows no band on the agarose gel (step 61–63).

### Potential solution

Check cDNA quality and repeat cDNA synthesis or from transfection forward.

Consider using a different PCR kit and optimizing PCR conditions. If the wild-type exon is not expressed, consider re-designing the assay to include a larger intronic region.

### Problem 3

The electropherogram in fragment analysis always shows double peaks.

### Potential solution

Make sure to use the final PCR product with only one FAM-labeled primer.

### Problem 4

The RNA concentration is too low at the end of the RNA extraction.

### Potential solution

Always optimize transfection conditions for the cell line of use, e.g. by increasing or decreasing incubation time after transfection or by increasing or decreasing the concentration of transfected plasmid.

## Resource availability

### Lead contact

Further information and requests for resources should be directed to the lead contact, Barbara Vona (barbara.vona@med.uni-goettingen.de).

### Technical contact

Questions about the technical specifics of performing the protocol should be directed to and will be answered by the technical contact, Hannah Andreae (hannah.andreae@stud.uni-goettingen.de).

### Materials availability

No new unique reagents were generated in this study. However, further requests for resources should be directed to the lead contact, Barbara Vona (barbara.vona@med.uni-goettingen.de).

### Data and code availability

This study did not generate code. Sequencing data and a dataset generated with this protocol is included.

## Acknowledgments

We are thankful to the 10.13039/501100001659German Research Foundation DFG VO 2138/7-1 grant 469177153, the 10.13039/501100001659DFG Heisenberg program VO 2138/8-1 grant 543719215, and the 10.13039/501100001659DFG Collaborative Research Center 1690 (Project A03 to B.V.), which supported this work. H.A. was funded through the Promotionskolleg scholarship funded by the Jacob-Henle-Programm and the Else Kröner-Fresenius-Stiftung at the University Medical Center of Göttingen. M.C. was funded by an Erasmus+ Traineeship grant from the 10.13039/100007195University of Naples Federico II (Università degli Studi di Napoli Federico II). We thank Dayana Warnecke at the Department of Molecular Neurobiology, Max Planck Institute for Multidisciplinary Sciences, for excellent technical support. B.V. is a member of the European Reference Network on Rare Congenital Malformations and Rare Intellectual Disability ERN-ITHACA (EU Framework Partnership Agreement ID: 3HP-HP-FPA ERN-01-2016/739516). This work was done with support of the Center for Rare Hearing Disorders at the Center of Rare Diseases Göttingen (ZSEG).

## Author contributions

H.A. and F.B. performed the experiments. H.A., M.C., D.O., S.E., and F.J. were involved in methodology validation and optimization. F.B., N.B., and B.V. supervised the study. H.A. wrote the first draft. All authors participated in review and editing. B.V. conceived the project and designed the experiment. All authors have read and agreed to the content.

## Declaration of interests

The authors declare no competing interests.
