# Supplementary material for: Protocol for a minigene splice assay using the pET01 vector
Source: STAR Protoc. 2025 Jun 18;6(3):103908. doi: 10.1016/j.xpro.2025.103908 (PMC12221276; doi:10.1016/j.xpro.2025.103908)
Supplement: Document S1. Figures S1–S4, Table S1, and Data S1 [file mmc1.pdf]

Data S1: Full plasmid pET01 sequence, related to before you begin.

Color legend:
Exon A and B of pET01
pET01 PCR primer 02 F: with BamHI restriction site
pET01 PCR primer 03 R: with SmaI restriction site
cDNA primer 01
XhoI restriction site
BamHI restriction site
SalI restriction site
NotI restriction site
ApaI restriction site
SmaI restriction site
SpeI restriction site
XbaI restriction site
SacII restriction site

AACGCCAGCAACGGAGATGCGCCGCGTGCGGCTGCTGGAGATGGCGGACGCGATGGATATGTT
CTGCCAAGGGTTGGTTTGCGCATTCACAGTTCTCCGCAAGAATTGATTGGCTCCAATTCTTGGAG
TGGTGAATCCGTTAGCGAGGTGCCGCCGGCTTCCATTCAGGTCGAGGTGGCCCGGCTCCATGC
ACCGCGACGCAACGCGGGGAGGCAGACAAGGTATAGGGCGGCGCCTACAATCCATGCCAACCC
GTTCCATGTGCTCGCCGAGGCGGCATAAATCGCCGTGACGATCAGCGGTCCAATGATCGAAGTT
AGGCTGGTAAGAGCCGCGAGCGATCCTTGAAGCTGTCCCTGATGGTCGTCATCTACCTGCCTGG
ACAGCATGGCCTGCAACGCGGGCATCCCGATGCCGCCGGAAGCGAGAAGAATCATAATGGGGA
AGGCCATCCAGCCTCGCGTCGGGGAGCTTTTTGCAAAAGCCTAGGCCTCCAAAAAAGCCTCCTC
ACTACTTCTGGAATAGCTCAGAGGCCGAGGCGGCCTCGGCCTCTGCATAAATAAAAAAAATTAGT
CAGCCATGGGGCGGAGAATGGGCGGAACTGGGCGGAGTTAGGGGCGGGATGGGCGGAGTTAG
GGGCGGGACTATGGTTGCTGACTAATTGAGATGCATGCAAGGAGATGGCGCCCAACAGTCCCCC
GGCCACGGGGCCTGCCACCATACCCACGCCGAAACAAGCGCTCATGAGCCCGAAGTGGCGAGC
CCGATCTTCCCCATCGGTGATGTCGGCGATATAGGCGCCAGCAACCGCACCTGTGGCGCCGGT
GATGCCGGCCACGATGCGTCCGGCGTAGAGGATCTCAGGATATAGTAGTTTCGCTTTTGCATAG
GGAGGGGGAAATGTAGTCTTATGCAATACTCTTGTAGTCTTGCAACATGCTTATGTAACGATGAGT
TAGCAACATGCCTTATAAGGAGAGAAAAAGCACCGTGCATGCCGATTGGTGGGAGTAAGGTGGT
ATGATCGTGGTATGATCGTGCCTTGTTAGGAAGGCAACAGACGGGTCTAACACGGATTGGACGA
ACCACTGAATTCCGCATTGCAGAGATATTGTATTTAAGTGCCTAGCTCGATACAATAAACGCCATT
TGACCATTCACCACATTGGTGTGCACCTCAAGCTTCCTGCATGCTGCTGCTGCTGCTGCTGCTGG
GCCTGAGGCTACAGCTCTCCCTGGGCATCATCCCAGTTGAGGAGGAGAACCCGGACTTCTGGAA
CCGCGAGGCAGCCGAGGCCCTGGGTGCCGCCAAGAAGCTGCAGCCTGCACAGACAGCCGCCA
AGAACCTCATCATCTTCCTGGGCGATGGGATGGGGGTGTCTACGGTGACAGCTGCCAGGATCGA
TCCGCTTCCTGCCCCTGCTGGCCCTGCTCATCCTCTGGGAGCCCCGCCCTGCCCAGGCTTTTGT
CAAACAGCACCTTTGTGGTTCTCACTTGGTGGAAGCTCTCTACCTGGTGTGTGGGGAGCGTGGA
TTCTTCTACACACCCATGTCCCGCCGCGAAGTGGAGGACCCACAAGGTAAGCTCTGCTCCTGAAT
TAATTCTATCCCAAGTGCTAACTACCCTGTTTGTCTTTCACCCTTGAGACCTTGTAAATTGTGCCCT
AGGTGTGGAGGGTCTCAGGCTAACCAGTGGGGGGCACATTTCTGTGGGCAGCTAGACATATGTA
AACATGGTAGCTGCCAGGAAGGAGTGAGAATCCTTCCTTAAGTCTCCTAGGTGGTGACGGGTGG
CTAGGCCCCAGGATAGGTACCGGGCCCCCCCTCGAGGTCGACGGTATCGATAAGCTAATTCCTG
CAGCCCGGGGGATCCACTAGTTCTAGAGCGGCCGCCACCGCGGTGGAGCTCGGTACCTATTTG
GGGACCCCATAGAGCACTGCACTGACTGAGGGATGGTAACAGGATGTGTAGGTTTTGGAGGCCC
ATATGTCCATTCATGACCAGTGACTTGTCTCACAGCCATGCAACCCTTGCCTCCTGTGCTGACTTA
GCAGGGGATAAAGTGAGAGAAAGCCTGGGCTAATCAGGGGGTCGCTCAGCTCCTCCTAACTGGA
TTGTCCTATGTGTCTTTGCTTCTGTGCTGCTGATGCTCTGCCCTGTGCTGACATGACCTCCCTGG
CAGTGGCACAACTGGAGCTGGGTGGAGGCCCGTGACCTTCAGACCTTGGCACTGGAGGTGGCC
CGGCAGAAGCGCG<u>GCATCGTGGATC</u>AGTGCTGCACCAGCATCTGCTCTCTCTACCAACTGGAGA
ACTACTGCAACTAGGCCCACCACTACCCTGTCCACCCCTCTGCAATGAATAAAACCTTTGAAAGA
GCACTACAAGTTGTGTGTACATGCGTGCATGTGCATATGTGGTGCGGGGGGAACATGAGTGGGG
CTGGCTGGAGTGGCGATGATAAGCTGTCAAACATGAGAATTCTTGAAGACGAAAGGGCCTCGTG
ATACGCCTATTTTTATAGGTTAATGTCATGATAATAATGGTTTCTTAGACGTCAGGTGGCACTTTTC
GGGGAAATGTGCGCGGAACCCCTATTTGTTTATTTTTCTAAATACATTCAAATATGTATCCGCTCA
TGAGACAATAACCCTGATAAATGCTTCAATAATATTGAAAAAGGAAGAGTATGAGTATTCAACATTT
CCGTGTCGCCCTTATTCCCTTTTTTGCGGCATTTTGCCTTCCTGTTTTTGCTCACCCAGAAACGCT
GGTGAAAGTAAAAGATGCTGAAGATCAGTTGGGTGCACGAGTGGGTTACATCGAACTGGATCTCA

ACAGCGGTAAGATCCTTGAGAGTTTTCGCCCCGAAGAACGTTTTCCAATGATGAGCACTTTTAAA
GTTCTGCTATGTGGCGCGGTATTATCCCGTGTTGACGCCGGGCAAGAGCAACTCGGTCGCCGCA
TACACTATTCTCAGAATGACTTGGTTGAGTACTCACCAGTCACAGAAAAGCATCTTACGGATGGCA
TGACAGTAAGAGAATTATGCAGTGCTGCCATAACCATGAGTGATAACACTGCGGCCAACTTACTT
CTGACAACGATCGGAGGACCGAAGGAGCTAACCGCTTTTTTGCACAACATGGGGGATCATGTAA
CTCGCCTTGATCGTTGGGAACCGGAGCTGAATGAAGCCATACCAAACGACGAGCGTGACACCAC
GATGCCTGCAGCAATGGCAACAACGTTGCGCAAACTATTAACTGGCGAACTACTTACTCTAGCTT
CCCGGCAACAATTAATAGACTGGATGGAGGCGGATAAAGTTGCAGGACCACTTCTGCGCTCGGC
CCTTCCGGCTGGCTGGTTTATTGCTGATAAATCTGGAGCCGGTGAGCGTGGGTCTCGCGGTATC
ATTGCAGCACTGGGGCCAGATGGTAAGCCCTCCCGTATCGTAGTTATCTACACGACGGGGAGTC
AGGCAACTATGGATGAACGAAATAGACAGATCGCTGAGATAGGTGCCTCACTGATTAAGCATTGG
TAACTGTCAGACCAAGTTTACTCATATATACTTTAGATTGATTTAAAACTTCATTTTTAATTTAAAAG
GATCTAGGCTGCTGCTTGCAAACAAAAAAACCACCGCTACCAGCGGTGGTTTGTTTGCCGGATCA
AGAGCTACCAACTCTTTTTCCGAAGGTAACTGGCTTCAGCAGAGCGCAGATACCAAATACTGTCC
TTCTAGTGTAGCCGTAGTTAGGCCACCACTTCAAGAACTCTGTAGCACCGCCTACATACCTCGCT
CTGCTAATCCTGTTACCAGTGGCTGCTGCCAGTGGCGATAAGTCGTGTCTTACCGGGTTGGACTC
AAGACGATAGTTACCGGATAAGGCGCAGCGGTCGGGCTGAACGGGGGGTTCGTGCACACAGCC
CAGCTTGGAGCGAACGACCTACACCGAACTGAGATACCTACAGCGTGAGCTATGAGAAAGCGCC
ACGCTTCCCGAAGGGAGAAAGGCGGACAGGTATCCGGTAAGCGGCAGGGTCGGAACAGGAGAG
CGCACGAGGGAGCTTCCAGGGGGAAACGCCTGGTATCTTTATAGTCCTGTCGGGTTTCGCCACC
TCTGACTTGAGCGTCGATTTTTGTGATGCTCGTCAGGGGGGCGGAGCCTATGGAAA

Table S1: Minigene assay primers designed for this protocol, related to before you begin.

Region of interest	Primer name	Primer sequence 5´- 3´	Product size
Exon 12	OTOF_Ex12 XhoI F	aattctcgagAGGGACCAAGACAGCATTTG	454 bp
	OTOF _Ex12 BamHI R	attggatccTTCTCAGCCATCCTCCATCA	
Intron 12 c.1205+2T>C	OTOF_Ex12_ c.1205+2T-C_mut_F	CATTGAGGGGcGAGGCCCAGC	--
	OTOF_Ex12_ c.1205+2T-C_mut_R	TCATCTTCGTCGGTCTCATTGG	
Exon 13	hu_OTOF_Ex13_XhoI_F	aattctcgagTGTTCTTGGGAGGTGGGTAT	553 bp
	hu_OTOF_Ex13_BamHI_R	attggatccTGGCCAACATGATTTCCAG	--
Intron 13 c.1392+1del	OTOF_Ex13_c.1392+1del_mut_F	TACTGGGGTATGAGGTAC	
	OTOF_Ex13_c.1392+1del_mut_R	CTTCTGGCCAGCAAAG	
Exon 28 +29	OTOF_Ex28+29 XhoI F	aattctcgagAATCTGGGGTGAACTACTGCC	693 bp
	OTOF _Ex28+29 BamHI R	attggatccTGACCTTCTTCTATGCCCACC	
Intron 28 c.3570+2T>C	OTOF_c.3570+2T>C_Ex28_mut_F	TTTGAAGTGGcGAGTGCAGGC	--
	OTOF_c.3570+2T>C_Ex28_mut_R	CCACTTGACGAGGGTG	
Exon 30	hu_OTOF_Ex30_XhoI_F	aattctcgagCGCTGGTTGATGGAGAAGA	676 bp
	hu_OTOF_Ex30_BamHI_R	attggatccATACGCATGCACGCACAA	
Intron 30 c.3864+1G>A	OTOF_c.3864+1G>A_Ex30_mut_F_2nd	GCTGGACGCGaTAAGGCGGGT	--
	OTOF_c.3864+1G>A_Ex30_mut_R_2nd	TTCACCATGGTCTCCAGTTTCTTGATGG	

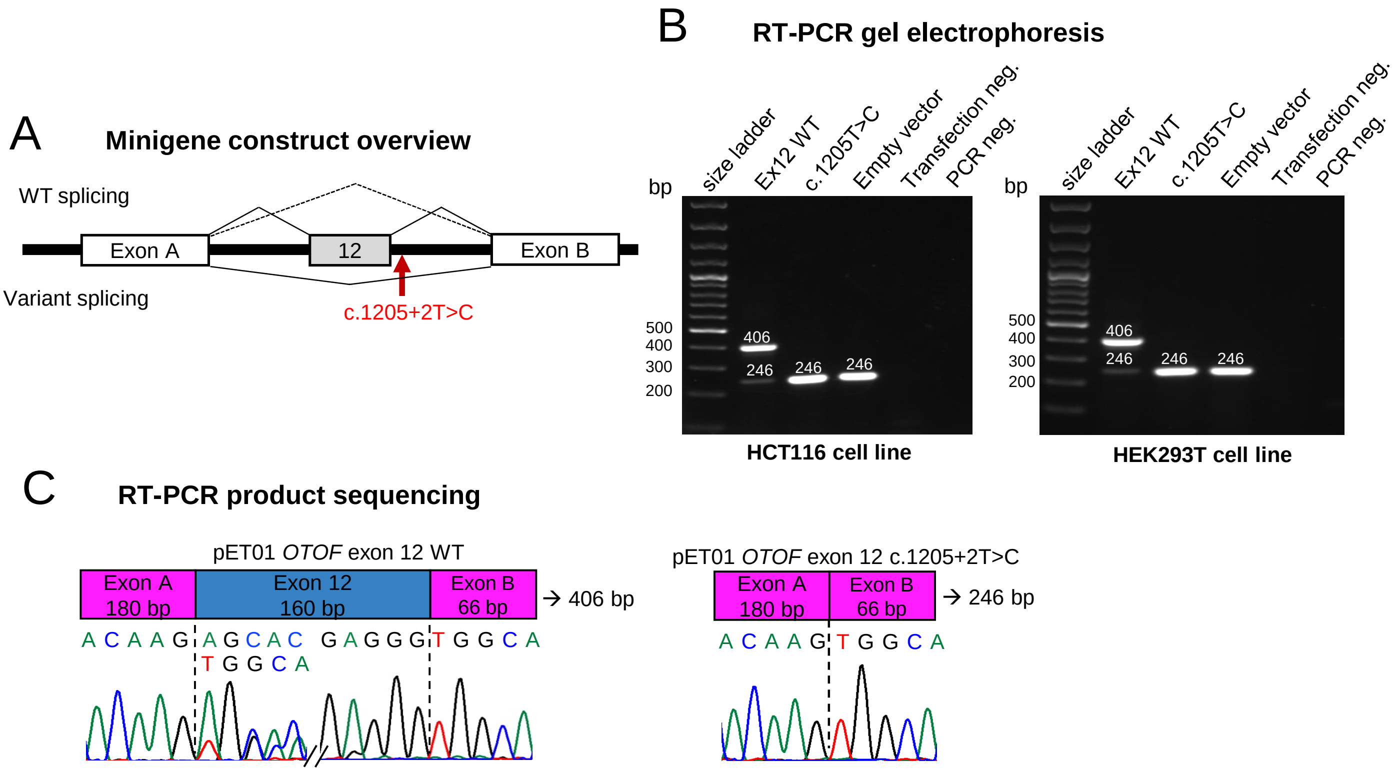

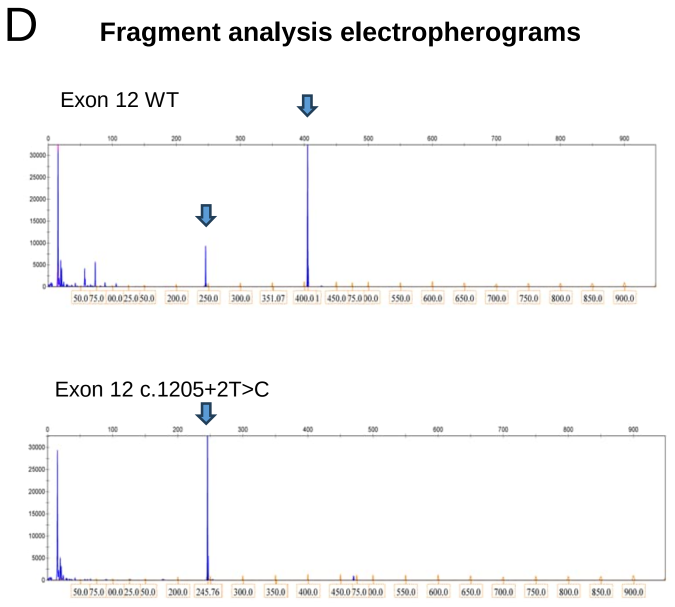

E Fragment analysis values

Exon 12 WT

Size (bp)	Mean actual size (bp)	Mean AUC	Percentage	Splicing pattern
HCT116 cells				
246	245.83	53839.33	17%	Exon 12 skipping
246	246.78	5788.33		
406	405.04	289429.67	83%	WT
HEK293T cells				
246	245.80	75873.67	19%	Exon 12 skipping
246	246.76	7728.67		
406	404.94	351337.33	80%	WT
	426.61	2511.00	1%	unknown

Exon 12 c.1205+2T>C

Size (bp)	Mean actual size (bp)	Mean AUC	Percentage	Splicing pattern	Consequence
HCT116 cells					
246	245.59	294944.00	100%	Exon 12 skipping	NM_001287489.2: r.(1046_1205del), p.(Glu349Glyfs*20)
246	246.77	37939.67			
HEK293T cells					
246	245.70	332691.67	100%	Exon 12 skipping	NM_001287489.2: r.(1046_1205del), p.(Glu349Glyfs*20)
246	246.78	38382.00			

Figure S1: Results for the minigene assay of the c.1205+2T>C *OTOF* variant, related to expected outcomes. A) Minigene construct schematic with exon 12 of *OTOF* cloned in between exon A and B of the vector. The red arrow displays the position of the variant. The WT and variant construct splicing pattern is indicated on top and bottom, respectively. B) Final RT-PCR products with controls after gel electrophoresis. Minigenes were transfected into HCT116 (left) and HEK293T (right) cells. C) Direct Sanger sequencing results of WT and variant final RT-PCR products of HCT116 cells. The primer PCR 02 F, aligning to exon A of pET01 was used as a primer for sequencing. Exon A and B of the vector are indicated by pink boxes, exon 12 by a blue box. The wild-type construct sample shows a mixture of normal exon 12 splicing and skipping of exon 12. The variant sample shows exon 12 skipping only. D) Electropherogram of the final RT-PCR product derived from HCT116 cells. Arrows indicate peaks of 246 bp and 406 bp. E) Fragment analysis peak results of FAM-labeled RT-PCR products. Only peaks > 200 RFU of height between 245-1000 bp, which are present in all three triplicates were considered. The size and area under the curve (AUC) of the peaks at the same base pair-length of triplicate samples were averaged. Wild-type expression of exon 12 was completely abolished in the variant, with the potential consequence on RNA and protein listed.

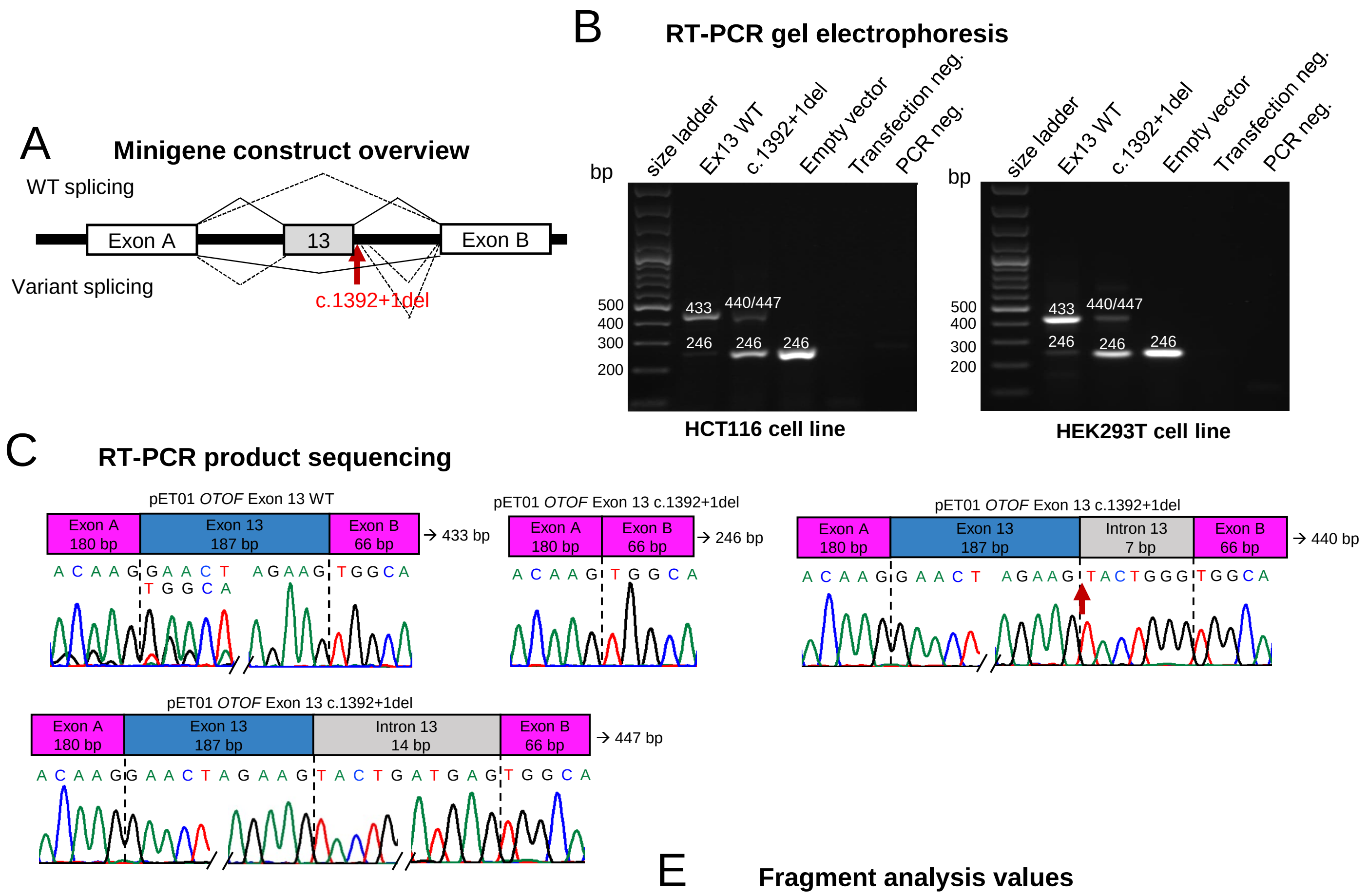

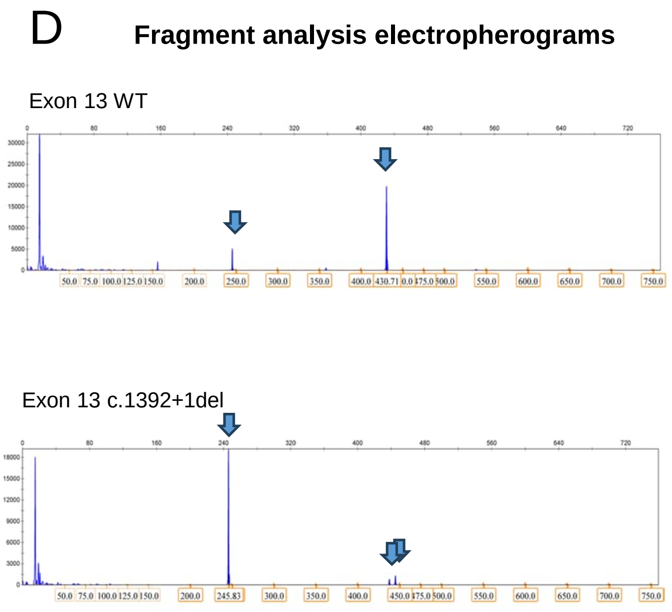

E Fragment analysis values

Exon 13 WT

Size (bp)	Mean actual size (bp)	Mean AUC	Percentage	Splicing pattern
HCT116 cells				
246	245.83	21502.67	11%	Exon 13 skipping
246	246.80	5719.67		
	357.86	3983.67	2%	unknown
433	430.51	197679.67	86%	WT
HEK293T cells				
246	245.86	43823.33	16%	Exon 13 skipping
246	246.78	3278.33		
	358.01	6274.33	2%	unknown
433	430.60	241073.00	81%	WT
	538.11	3714.67	1%	unknown

Exon 13 c.1392+1del

Size (bp)	Mean actual size (bp)	Mean AUC	Percentage	Splicing pattern	Consequence
HCT116 cells					
246	245.82	156111.25	56%	Exon 13 skipping	NM_001287489.2: r.(1207_1393del), p.(Asn403Alafs*31)
246	246.79	33068.33			
	372.20	2086.33	1%	unknown	
440	437.84	41954.33	12%	Activation of cryptic splice acceptor site (7 bp)	NM_001287489.2: r.(1392_1393insuacu ggg), p.(Gly465TyrfsTer13)
447	444.98	109404.67	32%	Activation of cryptic splice acceptor site (14 bp)	NM_001287489.2: r.(1392_1393insuacuggg guaugag), p.(Gly465TyrfsTer36)
HEK293T cells					
246	245.77	215991.33	86%	Exon 13 skipping	M_001287489.2: r.(1207_1393del), p.(Asn403Alafs*31)
246	246.79	18592.00			
440	437.86	14638.33	5%	Activation of cryptic splice acceptor site (7 bp)	NM_001287489.2: r.(1392_1393insuacuggg), p.(Gly465TyrfsTer13)
447	445.04	23575.67	9%	Activation of cryptic splice acceptor site (14 bp)	NM_001287489.2: r.(1392_1393insuacuggg guaugag), p.(Gly465TyrfsTer36)

Figure S2: Results for the minigene assay of the c.1392+1del *OTOF* variant, related to expected outcomes. (A) Minigene construct schematic with exon 13 of *OTOF* cloned in between exon A and B of the vector. The red arrow displays the position of the variant. The WT and variant construct splicing pattern is indicated on top and bottom, respectively. (B) Final RT-PCR products with controls after gel electrophoresis. Minigenes were transfected into HCT116 (left) and HEK293T (right) cells. (C) Direct Sanger sequencing results of WT and final RT-PCR products of HCT116 cells. The variant RT-PCR product was cloned for sequencing. The PCR 02 F primer, aligning to exon A of pET01 was used as a primer for sequencing. Exon A and B of the vector are indicated by pink boxes, exon 13 by a blue box and intronic sequence by grey boxes. The wild-type construct shows normal exon 13 splicing and skipping of exon 13. The variant sample shows exon 13 skipping, as well as activation of a 7 bp and 14 bp cryptic splice acceptor site. (D) Electropherogram of the final RT-PCR product derived from HCT116 cells. Arrows indicate peaks of 246 bp and 433 bp for WT and 246 bp, 440 bp and 447 bp for the variant. (E) Fragment analysis peak results of FAM-labeled RT-PCR product. Only peaks > 200 RFU of height between 245-1000 bp, which were present in all three triplicates were considered. The size and area under the curve (AUC) of the peaks at the same base pair-length of triplicate samples were averaged. Wild-type expression of exon 13 was completely abolished due to the variant, with the potential consequence on RNA and protein level included.

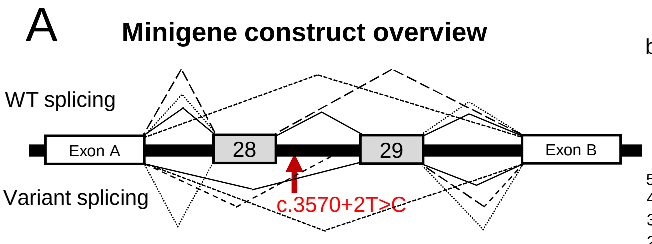

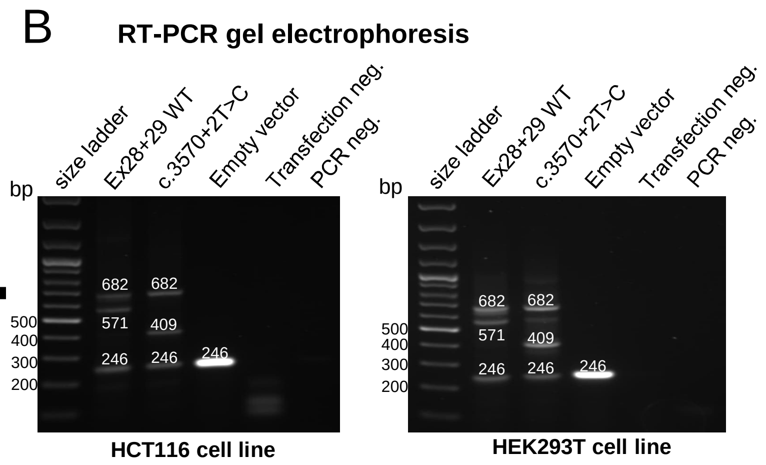

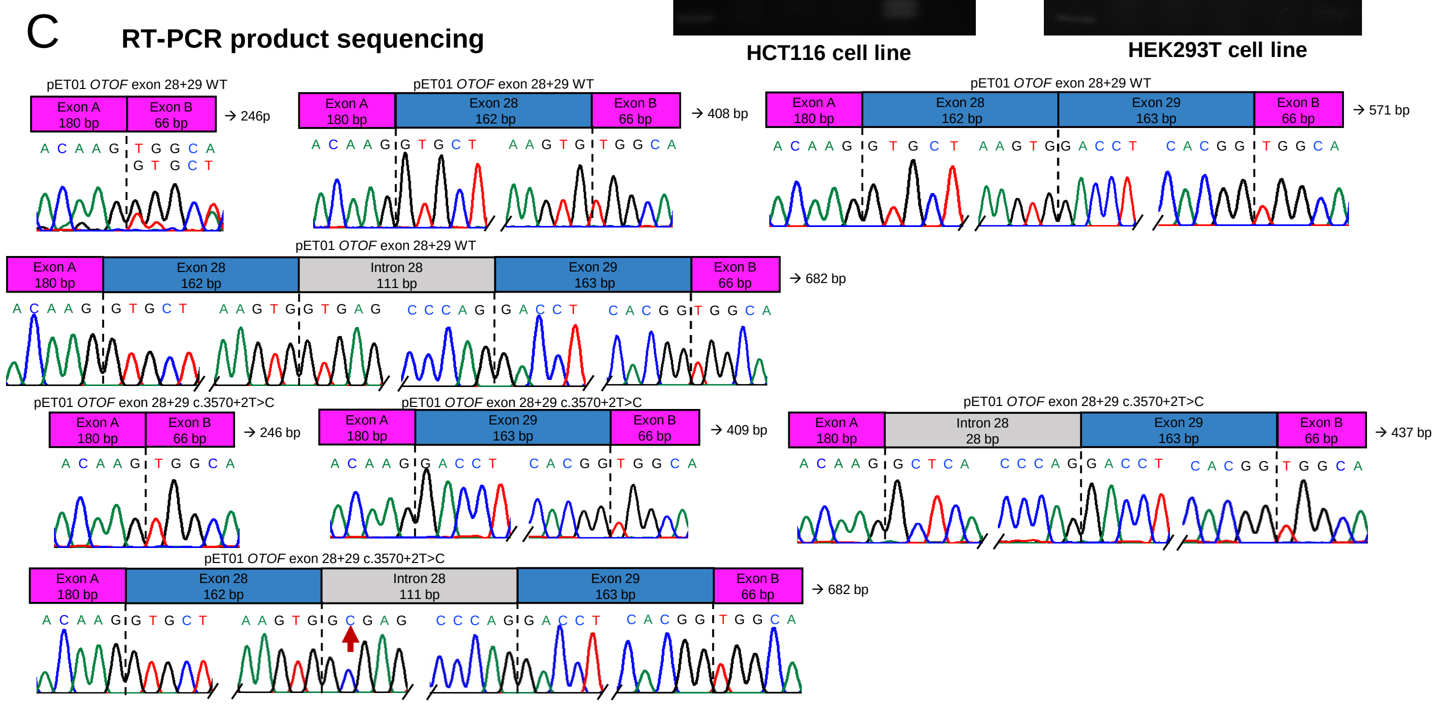

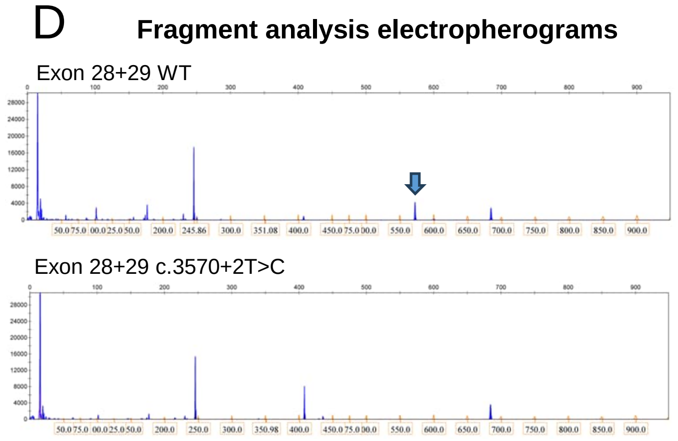

E Fragment analysis values

Exon 28+29 WT

Size (bp)	Mean actual size (bp)	Mean AUC	Percentage	Splicing pattern
HCT116 cells				
246	245.77	112673.00	56%	Exon 28+29 skipping
246	246.75	10718.67		
	250.20	5278.33	2%	unknown
	286.63	1521.33	1%	unknown
408	407.88	11865.33	5%	Exon 28 skipping
571	572.42	45776.67	21%	28+29 WT
682	684.72	34979.67	16%	Intron 28 retention
HEK293T cells				
246	245.82	105896.33	43%	Exon 28+29 skipping
246	246.79	8146.00		
	250.19	7991.33	3%	unknown
408	407.88	9026.33	3%	Exon 28 skipping
571	572.62	62046.33	23%	28+29 WT
682	684.91	72789.67	27%	Intron 28 retention

Exon 28+29 c.3570+2T>C

Size (bp)	Mean actual size (bp)	Mean AUC	Percentage	Splicing pattern	Consequence
HCT116 cells					
246	245.83	111317.00	44%	Exon 28+29 skipping	NM_001287489.2:r.(3409_3733del), p.(Val1137Serfs*25)
246	246.79	14043.33			
	250.20	5014.00	2%	unknown	
409	407.90	85926.67	30%	Exon 29 skipping	NM_001287489.2:r.(3571_3733del), p.(Asp1191Serfs*25)
	429.55	2655.00	1%	unknown	
437	435.52	9844.67	3%	Exon 28 skipping+activation of cryptic splice acceptor site (28bp)	NM_001287489.2:r.(3410_3569delinscucaggcuccugucgccaacacccca), p.(Val1137Alafs*110)
682	684.46	56648.67	20%	Intron 28 retention	NM_001287489.2:r.(3570_3571ins[gc;3570+3_3571-1), p.Val1190_Asp1191insX[37]
HEK293T cells					
246	246.80	6616.67	2%	Exon 28+29 skipping	NM_001287489.2:r.(3409_3733del), p.(Val1137Serfs*25)
	250.17	4024.33	1%	unknown	
409	407.92	113553.00	42%	Exon 29 skipping	NM_001287489.2:r.(3571_3733del), p.(Asp1191Serfs*25)
	429.63	3580.00	1%	unknown	
437	435.57	12956.33	5%	Exon 28 skipping+activation of cryptic splice acceptor site (28bp)	NM_001287489.2:r.(3410_3569delinscucaggcuccugucgccaacacccca), p.(Val1137Alafs*110)
682	684.58	53603.67	20%	Intron 28 retention	NM_001287489.2:r.(3570_3571ins[gc;3570+3_3571-1), p.Val1190_Asp1191insX[37]

Figure S3: Results for the minigene assay of the c.3570+2T>C *OTOF* variant, related to expected outcomes. (A) Minigene construct schematic with exon 28 and 29 of *OTOF* cloned in between exon A and B of the vector. The red arrow displays the position of the variant. The WT and variant construct splicing pattern is indicated on top and bottom, respectively. (B) Final RT-PCR products with controls after gel electrophoresis. Minigenes were transfected into HCT116 (left) and HEK293T (right) cells. (C) The final WT RT-PCR of HCT116 cells was initially directly Sanger sequenced and subsequently WT and variant samples were cloned for sequencing. The variant RT-PCR product was cloned for sequencing. The PCR 02 F primer, aligning to exon A of pET01 was used as a primer for sequencing. Exon A and B of the vector are indicated by pink boxes, exon 28 and 29 by a blue box and intronic sequence by grey boxes. The wild-type construct sample and the variant sample show complex splicing patterns. (D) Electropherogram of the final RT-PCR product derived from HCT116 cells. The arrow indicates the position of the 571 bp WT peak, which is missing in the variant. (E) Fragment analysis peak results of FAM-labeled RT-PCR product. Only peaks > 200 RFU of height between 245-1000 bp, which presented in all three triplicates were considered. The size and area under the curve (AUC) of the peaks at the same base pair-length of triplicate samples were averaged. Wild-type expression of both exons 28 and 29 was completely abolished in the variant. The potential consequence on RNA and protein level is also listed.

A Minigene construct overview

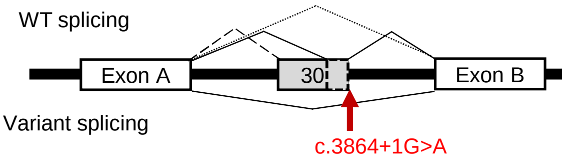

B RT-PCR gel electrophoresis

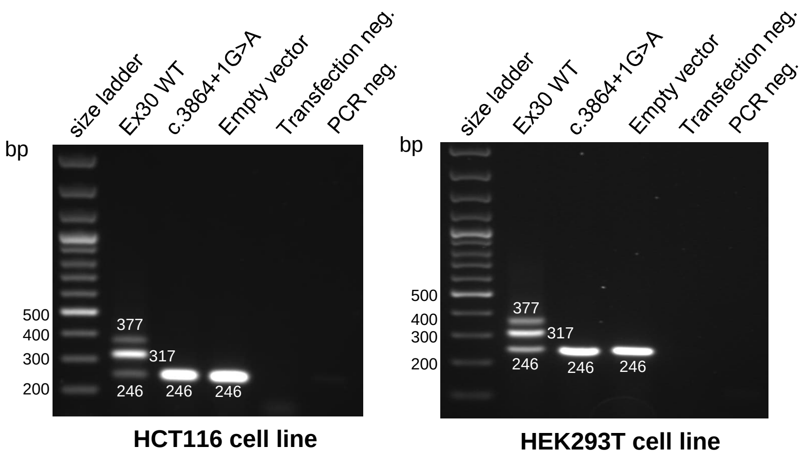

C RT-PCR product sequencing

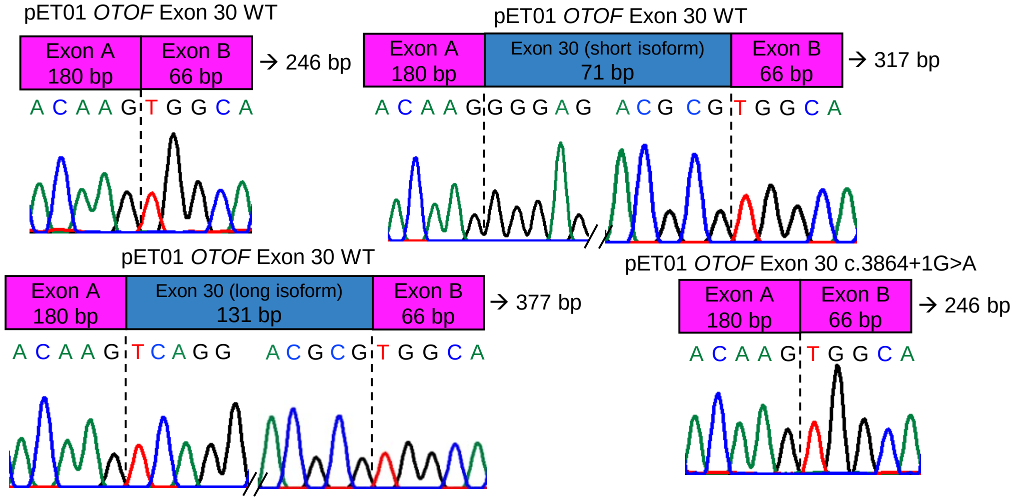

D Fragment analysis electropherograms

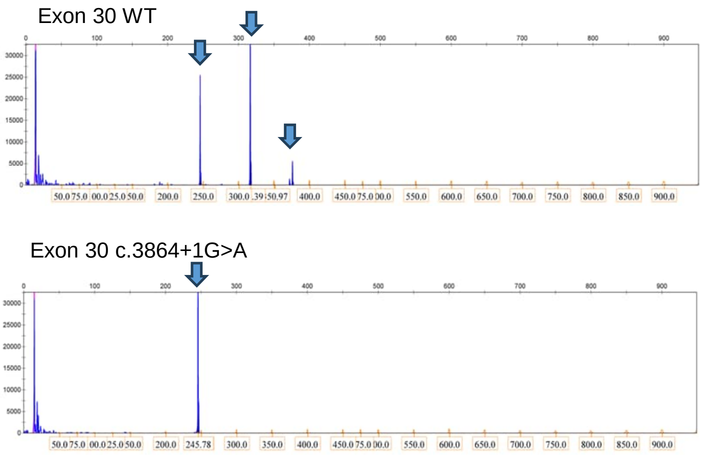

E Fragment analysis values

Exon 30 WT

Size (bp)	Mean actual size (bp)	Mean AUC	Percentage	Splicing pattern
HCT116 cells				
246	245.82	152573.33	31%	Exon 30 skipping
246	246.80	18474.67		
317	316.45	280947.00	58%	Short isoform exon 30 WT
317	317.52	37051.33		
	372.06	11934.67	2%	unknown
377	376.16	43853.33	9%	Long isoform exon 30 WT
377	377.06	5297.33		
HEK293T cells				
246	245.78	199937.33	46%	Exon 30 skipping
246	246.82	11516.00		
317	316.51	174006.67	40%	Short isoform exon 30 WT
317	317.50	9565.33		
	372.40	13858.67	3%	unknown
377	376.43	51442.00	11%	Long isoform exon 30 WT

Exon 30 c.3864+1G>A

Size (bp)	Mean actual size (bp)	Mean AUC	Percentage	Splicing pattern	Consequence
HCT116 cells					
246	245.56	355059.67	100%	Exon 30 skipping	NM_001287489.2: r.(3734_3864del), p.(Val1245Aspfs*3)
246	246.81	53192.67			
HEK293T cells					
246	245.72	283036.00	100%	Exon 30 skipping	NM_001287489.2: r.(3734_3864del), p.(Val1245Aspfs*3)
246	246.80	25477.33			

Figure S4: **Results for the minigene assay of the c.3864+1G>A *OTOF* variant, related to expected outcomes.** (A) Minigene construct schematic with Exon 30 of *OTOF* cloned in between exon A and B of the vector. The short isoform is indicated on the left of the dashed box. The red arrow displays the position of the variant. The WT and variant construct splicing pattern is indicated on top and bottom, respectively. (B) Final RT-PCR products after gel electrophoresis. Minigenes were transfected into HCT116 (left) and HEK293T (right) cells. (C) WT final RT-PCR products of HCT116 cells were cloned to sequence, while the variant sample was directly Sanger sequenced. The primer PCR 02 F, aligning to Exon A was used for sequencing. Exon A and B of the vector are indicated by pink boxes, exon 30 by a blue box. The wild-type construct sample shows normal exon 30 splicing (short and long isoform) and skipping of exon 30. The variant sample shows Exon 30 skipping only. (D) Electropherogram of the final RT-PCR product derived from HCT116 cells. Arrows indicate 246 bp, 317 bp and 377 bp. (E) Fragment analysis peak results of RT-PCR product from HCT116 cells. Only peaks > 200 RFU of height between 245-1000 bp, which presented in all three triplicates were considered. The size and area under the curve (AUC) of the peaks at the same base pair-length of triplicate samples were averaged. The potential consequence on RNA and protein level is also listed.
